# Improvement of Spatial Modeling of Cr, Pb, Cd, As and Ni in Soil Based on Portable X-ray Fluorescence (PXRF) and Geostatistics: A Case Study in East China

**DOI:** 10.3390/ijerph16152694

**Published:** 2019-07-28

**Authors:** Fang Xia, Bifeng Hu, Shuai Shao, Dongyun Xu, Yue Zhou, Yin Zhou, Mingxiang Huang, Yan Li, Songchao Chen, Zhou Shi

**Affiliations:** 1 Key Laboratory of Environment Remediation and Ecological Health, Ministry of Education, College of Environmental and Resource Sciences, Zhejiang University, Hangzhou 310058, China; 2 Sciences de la Terre et de l’Univers, Orléans University, 45067 Orléans, France; 3 Unité de Recherche en Science du Sol, INRA, 45075 Orléans, France; 4 InfoSol, INRA, US 1106, F-4075 Orléans, France; 5 Institute of Land Science and Property, School of Public Affairs, Zhejiang University, Hangzhou 310058, China; 6 Information Center of Ministry of Ecology and Environment, Beijing 100035, China

**Keywords:** heavy metals, Portable X-ray fluorescence, Co-Ordinary kriging, multi-variables indicator kriging, hot spots, multi-heavy metals pollution risk

## Abstract

To verify the feasibility of portable X-ray fluorescence (PXRF) for rapidly analyzing, assessing and improving soil heavy metals mapping, 351 samples were collected from Fuyang District, Hangzhou City, in eastern China. Ordinary kriging (OK) and co-ordinary kriging (COK) combined with PXRF measurements were used to explore spatial patterns of heavy metals content in the soil. The Getis-Ord index was calculated to discern hot spots of heavy metals. Finally, multi-variable indicator kriging was conducted to obtain a map of multi-heavy metals pollution. The results indicated Cd is the primary pollution element in Fuyang, followed by As and Pb. Application of PXRF measurements as covariates in COK improved model accuracy, especially for Pb and Cd. Heavy metals pollution hot spots were mainly detected in northern Fuyang and plains along the Fuchun River in southern Fuyang because of mining, industrial and traffic activities, and irrigation with polluted water. Area with high risk of multi-heavy metals pollution mainly distributed in plain along the Fuchun River and the eastern Fuyang. These findings certified the feasibility of using PXRF as an efficient and reliable method for soil heavy metals pollution assessment and mapping, which could contribute to reduce the cost of surveys and pollution remediation.

## 1. Introduction

Soil is critical to many ecosystem functions, and soil degradation and pollution have gained a great deal of attention worldwide [1,2,3,4,5,6]. Contaminated soils can pose serious threats to human health through a variety of pathways including diet, inhalation and dermal contact [7,8,9,10,11,12]. Soil pollution has become especially serious in China as a result of the rapid urbanization and industrialization that has occurred in the last several decades [13,14]. Indeed, a national survey report issued by the Ministry of Natural Resources and the Ministry of Ecology and Environment of the People’s Republic of China in 2014 revealed that the proportion of heavy metals contaminated soil samples in China was 16.1% [15].

Traditionally, soil heavy metals are measured by several analysis methods conducted in the laboratory, including atomic absorption spectrophotometry (AAS), atomic fluorescence spectrometry (AFS), inductively coupled plasma-atomic emission spectrometry (ICP-AES) and inductively coupled plasma-mass spectroscopy (ICP-MS) [16]. These methods have high accuracy and are widely used in many related studies [17,18,19,20]; however, they are complex, labor intensive, expensive, and require a long amount of time. In addition, the sampling process is destructive and results in discharge of waste that could lead to additional pollution. Polluted areas are usually represented by large spatial variations in the distribution of heavy metals, indicating that extensive surveys are necessary to capture spatial patterns of heavy metals in soil. This amplifies the shortcoming of laboratory analysis by requiring labor, time and additional costs, as well as intensive sampling. Therefore, quick and accurate alternative methods are urgently needed to replace the laboratory analysis methods described above. 

Proximal sensing by PXRF spectrometry could overcome the drawbacks of conventional laboratory analysis methods. Many studies have shown that PXRF could be a rapid and non-destructive measurement of heavy metals in soils. XRF, which is based on the excitation of inner electrons and the emission of photons after they relax to their ground state (fluorescence) [21], has been widely used to analyze heavy metals in soil. 

This rapid in situ analysis method also facilitates soil pollution assessment and mapping to identify polluted areas of interest for further remediation. Sacristán et al. (2016) [22] demonstrated the feasibility of PXRF for rapid assessment of Cu concentrations in agricultural soil and lettuce (*Lactuca sativa* L.) in Ginninderra Australia. Wan et al. (2019) [23] used PXRF to perform rapid assessment and mapping of heavy metals pollution in agricultural soils in Kunming, China. Brent et al. (2017) [24] validated the PXRF for in situ measurement of Hg in soils. Hu et al. (2017) [25] employed PXRF and Vis-NIR sensors to assess soil heavy metals pollution in Hangzhou, China. However, further application of PXRF has been deterred by drawbacks such as its high detection limit and low accuracy for certain heavy metals and because it is easily disturbed by environment conditions or other soil properties, such as water and pre-treatment steps. 

Co-kriging (COK) is the extension of ordinary kriging (OK) by incorporating auxiliary variables. The auxiliary variables should be more easily obtained than target variables [26,27]. The use of PXRF data as an auxiliary variable in COK mapping heavy metals distribution instead of direct use of PXRF data to employ interpolation is more promising than OK interpolation based on laboratory measurements or PXRF estimation values, because it can include more information to facilitate spatial estimation of soil heavy metals. However, few studies of the use of PXRF data as covariates during mapping of soil heavy metals have been conducted to date. Kim et al. [28] used PXRF measurement as covariates during analysis of the spatial distribution of As and Pb in soils in a former smelting area in South Korea and found that PXRF could reduce the effort required for collection of soil samples for conventional analysis and improve spatial estimations of polluted areas. However, to the best of our knowledge, few studies have used data from PXRF measurements as covariates to estimate spatial distributions of Cr, Pb, Cd, As and Ni in soil with COK to date [23,26,27,28]. 

Therefore, the present study was conducted to: (1) assess Cr, Pb, Cd, As and Ni contamination in soil in the survey area; (2) evaluate the feasibility of using XRF data as auxiliary variables for estimation of the aforementioned metals in soils across the study area; and (3) identify pollution hot spots and explore multi-heavy metals pollution risks across the study area.

## 2. Materials and Methods

### 2.1. Study Area and Soil Sampling

**Figure 1:**
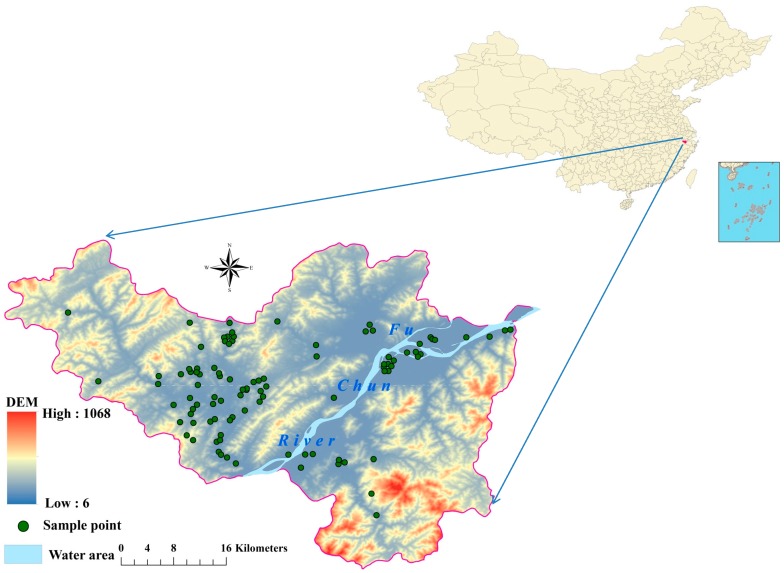
Location of samples in study area.

The study was conducted in the Fuyang district situated in Hangzhou, Zhejiang Province, eastern China (Figure 1). The Fuyang district covers an area of 1821 km^2^ (29°44′4″–30°11′58.5″ N and 119°25′00″–120°19′30″ E) and is characterized by its economic development, especially with respect to mining and industry, since the 1980s. However, this rapid economic growth has led to increased environmental threats, including soil degradation and soil heavy metals pollution. A more detailed description of Fuyang is provided in our previous study [25]. A total of 351 topsoil samples (0–20 cm) were collected from arable land in the study area in 2013. At each sampling site, five individual samples were collected using a random sampling design within a 10 × 10 m area. These subsamples were then mixed to obtain an integrated sample for each location. 

### 2.2. Samples Analyses

#### 2.2.1. ICP-AES

Soil sample analyses were conducted according to related national standards [29,30] issued by the Ministry of Ecology and Environment of the People’s Republic of China. Soil pH was determined using a pH meter to analyze a water:soil mixture at a ratio of 2.5:1. Concentrations of Cr, Pb, Cd, As and Ni in all 351 soil samples were determined by the ICP-AES method in the laboratory after dissolution with hydrochloric acid, hydrofluoric acid, nitric acid and perchloric acid.

#### 2.2.2. PXRF

The calibration dataset (N = 97) was scanned using a Niton XL2 GOLDD XRF Analyzer (Thermo Fisher Scientific Inc., Waltham, MA, USA) to determine the Cr, Pb, Cd, As and Ni concentrations. The instrument was calibrated before measurement and then corrected every 30 samples. The instrument was equipped with an Ag anode operating at a maximum of 45 kV and 80 μA. Before analysis, ground soils were put in a 31 mm X-ray sample cup and then covered with an X-ray film. Three parallel samples were scanned for each soil sample and the averaged measurement values were used to represent the content of heavy metals of soil samples. The time needed for each measurement was 90 s.

### 2.3. Pollution Assessment

The single pollution index (SPI) was calculated to determine contamination levels of individual elements and the Nemerow composite pollution index (NCPI) was employed to evaluate overall heavy metals pollution status in soils in the study area [31]. The details are provided in the Supplementary Material.

### 2.4. Geostatistics Models

#### 2.4.1. Ordinary Kriging (OK)

Ordinary kriging is one of the most widely used univariate interpolation methods [32]. In this study, OK was used to identify the spatial pattern of heavy metals content in the survey region. Experimental semi-variograms were calculated to indicate the spatial dependence of soil heavy metals using the following equation [32]:

(1) $$\gamma^{*}\left(h\right)=\frac{1}{2N\left(h\right)}\overset{N\left(h\right)}{\underset{i=1}{\sum }}{\left[Z\left(x_{i}\right)-Z\left(x_{i}+h\right)\right]}^{2}$$

where γ*(*h*) represents the semi-variance, *N*(*h*) indicates the number of separated experimental point pairs at distance lag *h*, *Z*(*x_i_*) indicates the measured value at observation site *i*, and *Z*(*x_i_* + *h*) expresses the measured value at observation site *i* + *h*. Based on the experimental variogram function, we can fit a suitable model using the weighted least squares and set some prior values for model parameters such as range, nugget, and sill prior during the process of fitting and interpolation.

#### 2.4.2. Co-Ordinary Kriging (COK)

COK is a multivariate extension of ordinary kriging that can utilize auxiliary variables and increase interpolation accuracy [33]. In COK, a cross-variogram is fitted to extract additional correlated information between target variables and auxiliary variables to obtain a more accurate estimation map. The cross-variograms were determined as previously described [34]:

(2) $$\gamma_{ij}\left(h\right)=\frac{1}{2N\left(h\right)}\overset{Z\left(h\right)}{\underset{a=1}{\sum }}\left[Z_{i}\left(x_{a}\right)-Z_{i}\left(x_{a}+h\right)\right]\;\left[Z_{j}\left(x_{a}\right)-Z_{j}\left(x_{a}+h\right)\right]$$

where *γ_ij_*(*h*) represents the cross-semivariogram function of target variables and auxiliary variables, h indicates the distance lag, and *N*(*h*) is the number of pairs of *Z_i_*(*x*) and *Z_j_*(*x*) at a separate distance h [35]. In this study, heavy metals content measured by PXRF was used as auxiliary information to employ co-ordinary kriging. The geostatistical interpolation was conducted with ArcGIS 10.2 (ESRI, ArcGIS 10.2, Redlands, CA, USA).

#### 2.4.3. Getis-Ord Index

The Getis-Ord spatial statistics index $\left(G_{i}^{*}\right)$ [36], which was issued by Ord and Getis, is able to detect statistically significant spatial clusters of high values (hot spots) and low values (cold spots) that enable it to identify spatial hotspots of heavy metals pollution. The Getis-Ord index has been described in detail in our previous study [37], and we have provided additional information regarding the index in the Supplementary Materials.

#### 2.4.4. Multi-Variables Indicator Kriging (MVIK)

The MVIK is an extension of univariate indicator kriging (IK) that converts a single continuous variable into binary data with a value of 0 or 1 based on fixed thresholds, and estimates the probability that the concentration of a heavy metal exceeds a specified threshold value at a given location [38]. The MVIK can combine the results of multiple thresholds into one comprehensive indicator and draw a comprehensive probability distribution map of heavy metal pollution [39,40].

The main steps of indictor kriging are as follows:

Define indicator codes for target variables:
  (3) $$I\left(x;z\right)=\{\begin{array}{ll} 1 & Z\left(x\right)\geq z\\ 0 & Z\left(x\right)<z \end{array}$$
  where *z* is the threshold and the observation values are transformed into a set of indicators. The probability of the target variable exceeding a fixed threshold was determined as follows:
  (4) $$Prob\left[z\left(x_{0}\right)\leq z_{k}/\left(n\right)\right]=\overset{n}{\underset{a=1}{\sum }}\lambda_{a}i\left(x_{a};z_{k}\right)$$
  where $i\left(x_{a};z_{k}\right)$ is the indicator value for the estimated variable $x_{a}\left(a=1,\ldots ,n\right)$, $\lambda_{a}$ is the weight for $i\left(x_{a};z_{k}\right)$ and the formula for calculating the weight is the same as that used for ordinary kriging.
The MVIK is the weighted results of several univariate indicator krigings:
  (5) $$I\left(x;z_{p}\right)=\overset{k}{\underset{i=1}{\sum }}w_{i}I\left(u;z_{i}\right)$$
  where, $I\left(x;z_{p}\right)$ is the comprehensive indicator value, $I\left(u;z_{i}\right)$ is the indicator value for the *i*th variable and $w_{i}$ is the weight for the *i* th variable. The weight is obtained by:
  (6) $$w_{i}=r_{i}/\overset{m}{\underset{i=1}{\sum }}r_{i}$$
  where *r_i_* is the toxicity response coefficient for the i th variable [37]. The toxicity response coefficients for Cd, As, Pb, Cr and Ni are 30, 10, 5, 2 and 5, respectively [41].

## 3. Results and Discussion

### 3.1. Summary of PXRF and ICP-AES Measurements

The summarized statistics for Cr, Pb, Cd, As and Ni content measured by ICP-AES and PXRF are provided in Table 1. Generally, the heavy metals contents determined by ICP-AES and PXRF were very consistent, indicating that PXRF showed a good ability to predict heavy metals contents in soil. The levels of Cd and As in soil in the study area were significantly higher than the background values in Zhejiang Province and China. The concentrations of Pb, Cr and Ni were lower than the corresponding background values in soil in Zhejiang province and China. 

**Figure 2:**
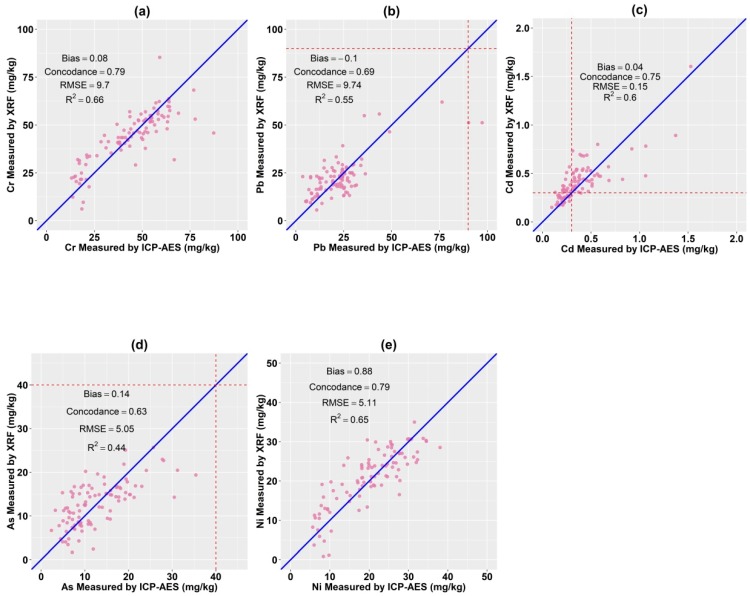
Scatter plot of Cr (**a**), Pb (**b**), Cd (**c**), As (**d**) and Ni (**e**) content measured by laboratory analysis (ICP-AES) and XRF (red dotted line represents the national limit value for corresponding heavy metals).

The heavy metals content measured by traditional laboratory analysis (ICP-AES) and PXRF is presented in Figure 2. R^2^ and concordance [44] indicates the correlation between the heavy metals content measured by PXRF and ICP-AES, whereas RMSE and bias represents the estimation accuracy. When compared with the results of ICP-AES analysis, strong linear correlations were detected in the PXRF measurements of heavy metals. As shown in Figure 2, the R^2^ values for Cr, Pb, Cd, Ni and As were 0.66, 0.55, 0.60, 0.65 and 0.44, respectively. And the RMSE for Cr, Pb, Cd, Ni and As equal to 9.77, 9.74, 0.15, 5.11 and 5.05, correspondingly. The concordance for different heavy metals were varied from 0.63 to 0.79 while the bias for different heavy metals varied between 0.04 to 0.88. These findings provide a good foundation for use of heavy metals content measured by PXRF as auxiliary data for COK.

### 3.2. Assessment of Soil Heavy Metals Pollution Status

The mean averaged single pollution index values for Cr, Pb, Cd, As and Ni of soil samples in the study area were 0.21, 0.24, 0.99, 0.41 and 0.27, respectively. The averaged pollution index for Cd was 0.99, which was almost at the level of slight pollution. The heavy metals pollution grades for soil samples classified by single pollution indexes are shown in Table 2. The Cr and Ni in all soil samples in the study area were at safe levels and showed no pollution. Additionally, 4.12%, 34.02% and 1.03% of the samples were polluted by As, Cd and Pb, respectively. The averaged composite pollution index for heavy metals in soil in the surveyed region was 0.78, which exceeded the alert limit. The heavy metals pollution grades for soil samples based on the composite pollution indexes are shown in Table 3. The composite pollution levels of nearly half of the soil samples were at or above the alert limit. Among these, 18.55% of soil samples showed slight, moderate or severe pollution.

Our results revealed the main pollutant element in the study area was Cd, followed by As and Pb. Fuyang is home to many smelting companies and mining activities. Although many mines have been closed in recent years, serious heavy metals pollution has long been detected in soil in Fuyang because of the long term waste discharge from mines [45,46,47]. In addition, Chen et al. (2013) [48] reported that application of the large amounts of chemical fertilizer contributed to heavy metals accumulation in the survey area. 

### 3.3. Spatial Modeling of Soil PTEs Based on Secondary Variables from Predicted Value of PXRF

#### 3.3.1. Spatial Pattern of Soil Heavy Metals Content

**Figure 3:**
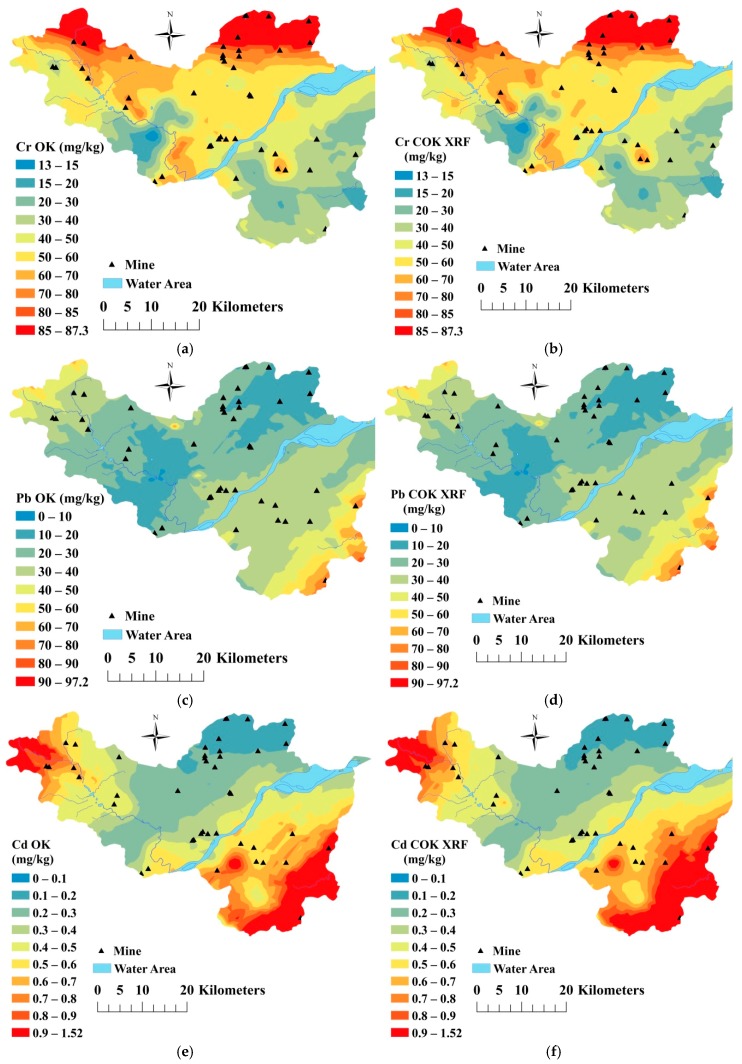

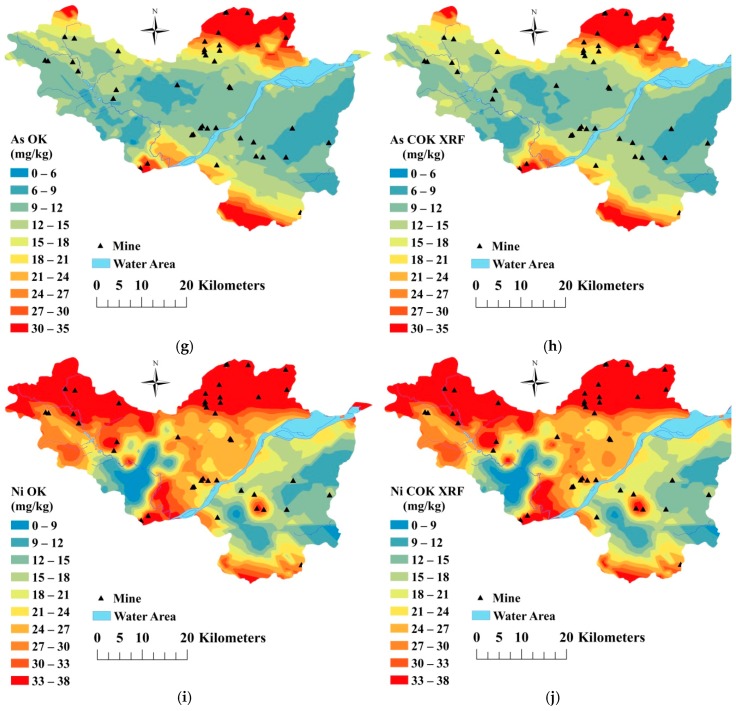
Spatial distribution of heavy metals content determined by ordinary kriging (OK): ((**a**) Cr, (**c**) Pb, (**e**) Cd, (**g**) As, (**i**) Ni) and co-ordinary kriging (COK): ((**b**) Cr, (**d**) Pb, (**f**) Cd, (**h**) As, (**j**) Ni).

The map of Cr, Pb, Cd, As and Ni content in soil generated by OK is presented in the left column of Figure 3, while the right column shows that obtained by COK. High values of Cr (Figure 3a,b) and Ni (Figure 3i,j) were mainly observed in the north and northwest part of the study area. The high values of Pb (Figure 3c,d) and Cd (Figure 3e,f) were mainly located in the southeast and northwest parts of the study area, while the high values of As (Figure 3g,h) were found in the north and south portions. The spatial patterns of heavy metals pollution produced by OK and COK were very similar, but COK captured more local variations, further confirming the advantage of COK over OK and that use of covariates in COK could provide more detailed information [28,49]. These results confirmed the conclusions of a previous study [22] that the application of XRF measurements as covariates could improve the spatial interpolation of soil heavy metals. 

#### 3.3.2. Model Accuracy

The accuracy of the OK and OCK methods was evaluated using validation data (N = 254) (Table 4). As described in Table 4, OK and COK showed the high accuracy for estimation of the spatial distribution of Cr, Cd and Ni content in soil and the accuracy for Pb and As was also acceptable. In particular, the R^2^ values of OK and COK interpolation for estimating the spatial distribution of As content in soil was 0.715 and 0.722, while they were 0.706 and 0.714 for Cr, respectively. 

The COK method, which used XRF measurement values as auxiliary data, obviously outperformed the OK method. When compared with the OK method, the R^2^ values of the COK interpolation for Pb, Cd, As, Cr and Ni improved by 12.72%, 10.10%, 6.67%, 1.13% and 0.98%, respectively, while the RMSE of the COK interpolation for Pb, Cd, As and Cr decreased by 4.54%, 7.70%, 2.32% and 0.68%, respectively, and that for Ni slightly increased by 0.22%. And the Concordance of the COK interpolation for Pb, Cd, As increased by 4.66%, 6.79%, 3.08%. The improvement of model accuracy for COK was affected by many factors, including the correlations between target variables and covariates [50], the spatial distribution of soil samples [50] and the number of soil samples [32]. A strong relationship between target variables and covariates is critical for COK [33]. In addition, the spatial coverage of soil samples also has important effects on model accuracy [50]. Webster et al. (2000) [32] reported that reliable semi-variance functions should be obtained from at least 100 samples. In our study, we utilized 97 soil samples for interpolation; however, this only meets the minimum requirements for the sample number.

#### 3.3.3. Hotspots of Soil Heavy Metals Pollution

**Figure 4:**
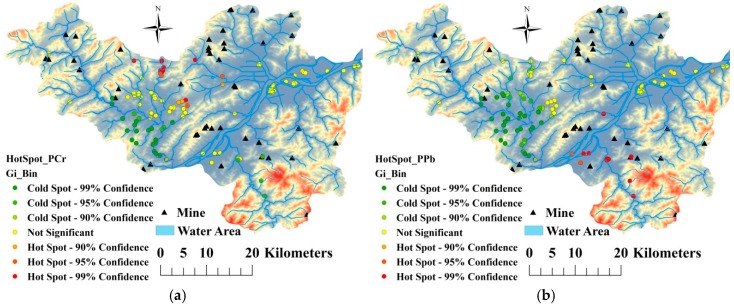

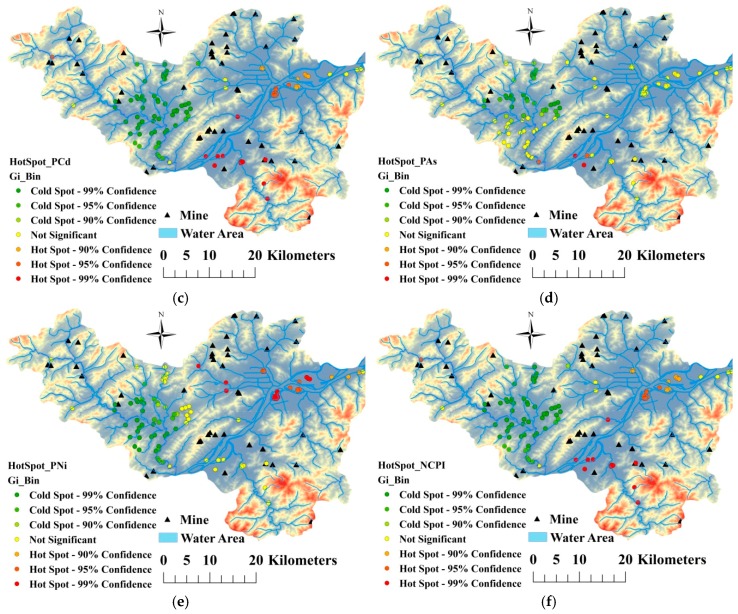
Hotspots of individual pollution indexes for Cr (**a**), Pb (**b**), Cd (**c**), As (**d**), Ni (**e**) and Nemerow composite pollution index (**f**).

In this study, the Getis-Ord index was calculated to detected hot spots of heavy metals pollution in soil (Figure 4). As shown in Figure 4, the hot spots of the pollution index for Cr in soil were mainly distributed in the northern part of Fuyang, while the hot spots of the pollution index for Pb and As were mainly located in southern Fuyang. The hot spots of the pollution index for Cd were located in plains along the Fuchun River, while those for Ni were located in plains around the upstream portion of the Fuchun River and those for the composite heavy metals pollution index values were mainly in the eastern part of Fuyang.

**Figure 5:**
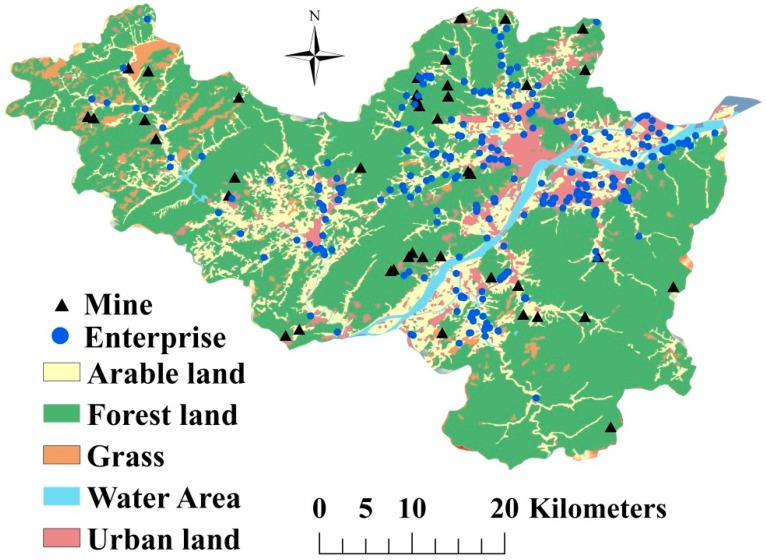
Land use map of Fuyang.

Overall, pollution hot spots of soil heavy metals in the study area were mainly distributed in northern Fuyang and in plains along the Fuchun River in southern Fuyang, with a portion of hot spots located in remote areas close to mines. When combined with the information provided in Figure 5, these findings indicate that the hot spots in the northern part of the study area were caused by anthropogenic activities such as industrial discharge, irrigation with polluted water in the Fuchun River, traffic emissions and mining activities. The hot spots in the southern part were caused by irrigation with polluted soil and mining activities.

### 3.4. Multi-Heavy Metals Pollution Risk in Study Area

**Figure 6:**
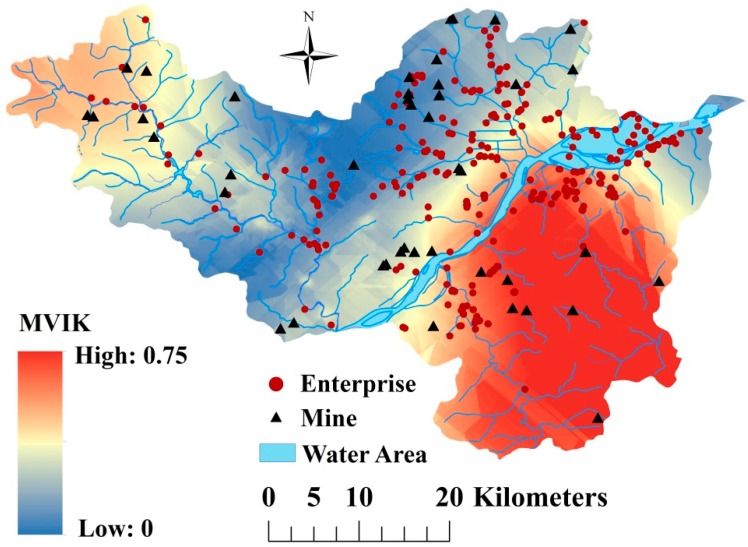
Spatial distribution of multi-heavy metals pollution risk produced by Multi-variables Indicator Kriging (MVIK).

The map of multi-heavy metals pollution index risk was obtained by MVIK and is shown in Figure 6. When compared with univariate indicator kriging, MVIK could provide more comprehensive results regarding overall heavy metal pollution risks in the study area. As shown in Figure 6, area along the Fuchun River and the eastern part of Fuyang there was a high risk of multi-heavy metals pollution indexes with a pollution risk higher than 0.5, with some areas having a risk as high as 0.75, indicating these area have a very high potential for soil heavy metal pollution. Most of hot spots of composite pollution index located in areas with high multi-heavy metals pollution risks. These findings confirmed the rationality of the results produced by MVIK. Notably, areas with high risks of multi-heavy metals pollution overlapped with areas of high population density in Fuyang. Because heavy metals pollution could lead to adverse effects on human health [7,14,37], measures are urgently needed to protect local residents from the negative health risks triggered by heavy metals pollution in soil.

## 4. Conclusions

Heavy metals pollution in soil is an issue of widespread concern in China that has led to an urgent need for rapid and accurate analysis and assessment of soil heavy metals pollution. PXRF was shown to be an alternative method to meet this demand and was employed in this study to assess heavy metals pollution and improve mapping of spatial distribution of heavy metals in the Fuyang District of Hangzhou in Zhejiang Province. Our results revealed that the content of Cd and As in soil was significantly higher than the background values in Zhejiang Province and China, and that Cd was the dominant pollution element in Fuyang. Moreover, the mean pollution index for Cd was 0.99, which is very close to the alert limit value for slight pollution. Furthermore, 34.02% of soil samples collected in Fuyang in this study were polluted by Cd. Estimation of the spatial distribution of heavy metals using PXRF measurements as auxiliary information in COK improved model accuracy, especially for Pb and Cd, when compared with OK. The pollution hot spots of heavy metals were mainly situated in northern Fuyang and the plains along the Fuchun River in southern Fuyang and were primarily a result of mining, industrial and traffic activities, as well as irrigation with polluted water. A map of the spatial distribution of multi-heavy metals pollution risk produced by MVIK showed high multi-heavy metals pollution index values in the area along the Fuchun River and the eastern part of the study area. In conclusion, PXRF is a reliable method that can rapidly and accurately determine levels of heavy metals such as Cr, Pb, Cd, As and Ni in soil. Moreover, this method can be used to improve the spatial interpolation accuracy of heavy metal contents when combined with COK. This could reduce labor as well as monetary and time costs when conducting soil heavy metals surveys, assessments and mapping. However, there are still several limitations of PXRF. Specifically, in-situ measurement of PXRF is easily affected by many factors including soil moisture and surface coverage of soil, which may lead to unstable measured results. Moreover, the detection limits of PXRF is higher than laboratory analysis methods such as ICP-AES, AAS, AFS and ICP-MS [23,25] which prevent its application for determining the content of heavy metals below the detection limits. 

## Figures and Tables

**Table 1:** Descriptive statistics of soil heavy metal concentrations measured by ICP-AES and PXRF (mg kg^−1^).

Statistics	Method	MEAN	SD	MIN	MAX	CV%	SBC_1_	SBC_2_
pH		5.8	1.1	3.8	8.01	18.9		
Cd	ICP-AES	0.37	0.24	0.1	1.53	64.81	0.07	0.097
	PXRF	0.41	0.2	0.15	1.6	48.65		
As	ICP-AES	12.59	6.77	2.36	35.39	53.73	9.2	11.2
	PXRF	12.73	5.11	1.69	25.68	40.17		
Pb	ICP-AES	22.68	14.64	3.59	97.24	64.53	23.7	26
	PXRF	22.59	10.12	5.63	62.01	44.79		
Cr	ICP-AES	43.79	16.86	13.1	87.32	38.5	52.9	61
	PXRF	43.86	13.64	6.14	85.34	31.1		
Ni	ICP-AES	20.31	8.27	5.62	38.02	40.74	24.6	26.9
	PXRF	21.18	8.01	0.86	52.03	37.8		

MEAN represents the averaged value; SD represents standard deviation; MIN represents minimum value; MAX represents maximum value; CV represents coefficient of variation; SBC_1_ represents the background content of heavy metals in soil in China [42]. SBC_2_ represents the background content of heavy metals in soil in Zhejiang Province [43]. ICP-AES represents inductively coupled plasma-atomic emission spectrometry; PXRF represents portable X-ray fluorescence.

**Table 2:** Heavy metals pollution grade classification based on the single pollution index (SPI).

Element		$P_{i}\leq 1$	$1<P_{i}\leq 2$	$2<P_{i}\leq 3$	$3<P_{i}\leq 5$	$5<P_{i}$
As	Sample Number	93	4	0	0	0
	Percentage	95.88%	4.12%	0	0	0
Cd	Sample Number	64	28	2	3	0
	Percentage	65.98%	28.86%	2.06%	3.09%	0
Cr	Sample Number	97	0	0	0	0
	Percentage	100%	0	0	0	0
Pb	Sample Number	96	1	0	0	0
	Percentage	98.97%	1.03%	0	0	0
Ni	Sample Number	97	0	0	0	0
	Percentage	100%	0	0	0	0

**Table 3:** Heavy metal pollution grade classification based on the Nemerow composite pollution index (NCPI).

Pollution Grade	Count	Proportion
Safety	52	53.61%
Alert	27	27.84%
Slight pollution	15	15.46%
Moderate pollution	2	2.06%
Severe pollution	1	1.03%

**Table 4:** Comparison of model accuracy of ordinary kriging (OK) and co-ordinary kriging (COK) for different heavy metals.

Element		R^2^	Concordance	RMSE
Cr	OK	0.706	0.83	8.76
	COK XRF	0.714	0.83	8.70
	Differences (%)	+1.13%	0	−0.68%
Pb	OK	0.456	0.655	8.60
	COK XRF	0.514	0.696	8.21
	Differences (%)	+12.72%	+4.66%	−4.54%
Cd	OK	0.624	0.766	0.13
	COK XRF	0.687	0.818	0.12
	Differences (%)	+10.10%	+6.79%	−7.70%
As	OK	0.450	0.65	4.75
	COK XRF	0.480	0.67	4.64
	Differences (%)	+6.67%	+3.08%	−2.32%
Ni	OK	0.715	0.84	4.64
	COK XRF	0.722	0.84	4.63
	Differences (%)	+0.98%	0	+0.22%

RMSE notes the Root Mean Square Error; PXRF notes portable X-ray fluorescence; COK: co-ordinary kriging; OK: ordinary kriging.

## Supplementary Materials

The following are available online at https://www.mdpi.com/1660-4601/16/15/2694/s1, Title: Single pollution and Nemero comprehensive pollution indices in soil; Sampling and chemical analysis.
