# Supplementary material for: Improvement of Spatial Modeling of Cr, Pb, Cd, As and Ni in Soil Based on Portable X-ray Fluorescence (PXRF) and Geostatistics: A Case Study in East China
Source: Int J Environ Res Public Health. 2019 Jul 28;16(15):2694. doi: 10.3390/ijerph16152694 (PMC6696468; doi:10.3390/ijerph16152694)
Supplement: Supplementary file 1 [file ijerph-16-02694-s001.pdf]

Single pollution and Nemero comprehensive pollution indices in soil

The degree of HM pollution in soil was assessed with reference to national regulations, and the pollution index was calculated to evaluate the quality of soil and investigate the impact of anthropogenic activities. The single pollution index (SPI) for each HM was determined (Equation (1)) followed by calculation of the Nemerow comprehensive pollution index (NCPI) (Equation (2)):

$$SPI = C_i/S_i \tag{1}$$

where C_i is the concentration of the HM in soil i and S_i is the pollution threshold of HM i in soil and:

$$NCPI = ((P_{imax})^2 + (\overline{SPI})^2)^{1/2} \tag{2}$$

where P_{imax} is the maximum SPI value of each HM and $\overline{P}$ is the mean SPI of each HM. As the NCPI is a comprehensive index, it was used to classify the soils in terms of HM pollution.

SPI is divided into four levels from no pollution to high pollution, and the degree of pollution is indicated fias follows: unpolluted ($SPI \le 1$), slightly polluted ($1 < SPI \le 2$), moderately polluted ($2 < SPI \le 3$), and highly polluted ($SPI > 3$). However, the classification of NCPI is slightly different from the SPI levels, and can be graded as safe (NCPI ≤ 0.7), precaution (0.7 < NCPI ≤ 1.0), slight pollution (1.0 < NCPI ≤ 2.0), moderate pollution (2.0 < NCPI ≤ 3.0) and heavy pollution (NCPI > 3.0).

Sampling and chemical analysis

A total of 351 topsoil samples (0–20 cm) were collected from arable land in the study area in 2013. At each sampling site, five individual samples were collected using a random sampling design within a 10 × 10 m area. These subsamples were then mixed to obtain an integrated sample for each location. All soil subsamples were collected using a stainless steel shovel. The sampling locations were accurately recorded using a differential global positioning system (GPS).

Soil samples were air-dried in a laboratory for several days at ambient temperature (~25 °C) and were passed through a 2 mm nylon sieve for general analysis of soil properties. Then, some of the soil samples were ground to pass through a 100-mesh screen (149 μm) and stored in closed polyethylene bags for analysis of HM concentrations. Soil sample analyses were conducted according to related national standards issued by the Ministry of Ecology and Environment of the People's Republic of China. Soil pH was determined using a pH meter to analyse a water:soil mixture at a ratio of 2.5:1. Concentrations of Cr, Pb, Cd, As and Ni in all 351 soil samples were determined by the ICP-AES method in the laboratory after dissolution with hydrochloric acid, hydrofluoric acid, nitric acid and perchloric acid. Reagent blanks and standard reference materials were used in the analysis for quality assurance and control. Element recoveries were in the 90–110% range.
